# *Mutator*-Like Elements with Multiple Long Terminal Inverted Repeats in Plants

**DOI:** 10.1155/2012/695827

**Published:** 2012-03-08

**Authors:** Ann A. Ferguson, Ning Jiang

**Affiliations:** Department of Horticulture, Michigan State University, East Lansing, MI 48824, USA

## Abstract

*Mutator*-like transposable elements (MULEs) are widespread in plants and the majority have long terminal inverted repeats (TIRs), which distinguish them from other DNA transposons. It is known that the long TIRs of *Mutator* elements harbor transposase binding sites and promoters for transcription, indicating that the TIR sequence is critical for transposition and for expression of sequences between the TIRs. Here, we report the presence of MULEs with multiple TIRs mostly located in tandem. These elements are detected in the genomes of maize, tomato, rice, and *Arabidopsis*. Some of these elements are present in multiple copies, suggesting their mobility. For those elements that have amplified, sequence conservation was observed for both of the tandem TIRs. For one MULE family carrying a gene fragment, the elements with tandem TIRs are more prevalent than their counterparts with a single TIR. The successful amplification of this particular MULE demonstrates that MULEs with tandem TIRs are functional in both transposition and duplication of gene sequences.

## 1. Introduction

Transposable elements (TEs) are DNA fragments that are capable of moving from one genomic location to another and increasing their copy numbers. Based on their transposition mechanisms, TEs fall into two classes: (1) Class I elements, or retrotransposons, that use the element-encoded mRNA as the transposition intermediate and (2) Class II elements, or DNA transposons, that transpose through a DNA intermediate. Autonomous transposons encode transposases that are responsible for the transposition of themselves and their cognate nonautonomous elements that do not encode transposases.

A common feature for DNA transposons, with a few exceptions, is the presence of a terminal inverted repeat (TIR) at each terminus of the element. As an essential structural component of the element, TIR plays important roles in transposition. For example, the transposase encoded by the bacterial *Tn3* element specifically binds to its TIR (38 bp in length), and this then facilitates the nicking at the end of *Tn3* by DNase I and initializes the transposition process [1]. In eukaryotes, it was shown that the transposase of the *Hermes* element binds to its imperfect TIRs and excises the element. This process is accompanied by the formation of a hairpin structure in the flanking donor sequence, resembling the V(D)J recombination process [2]. Binding of transposase to the TIR and to the target DNA mediates the synapsis of the transposon ends and the target DNA, allowing the insertion of the element into the target sequence [3]. The presence of the TIR sequence also influences the target specificity of the element. For instance, the deletion of a 4 bp sequence within the binding region of the TIR in *Tn3* abolishes its transposition immunity, that is, the phenomenon whereby *Tn3* avoids insertion into another *Tn3* element [4].

The TIRs of DNA transposons are usually less than 50 bp in length and can be as short as 8 bp [5]. Nevertheless, there are a few transposon families with exceptionally long TIRs and among these is the *Mutator* superfamily of transposable elements. First discovered in maize in 1978 [6], *Mutator *and *Mutator*-like elements (MULEs) appear to be prevalent among eukaryotes. Subsequent to the initial discovery in maize, MULEs have been found in other plant genomes such as *Arabidopsis*, rice, and* Lotus japonicus *[7–9], as well as in fungal and animal genomes [10, 11]. In addition to their unparalleled activity in maize [12], MULEs include a special subgroup of elements referred to as Pack-MULEs which are nonautonomous MULEs carrying genes or gene fragments. The sequence acquisition by Pack-MULEs may result in the formation of new open reading frames and the potential to regulate the expression of the parental genes from which the fragments are derived [8, 13, 14].

*Mutator* and MULEs are distinguished from other DNA transposable elements by having a 9–11 bp target site duplication (TSD) which flanks the element and are formed during transposition into a new genomic location. The TIRs of MULEs, typically ranging from 100 to 500 bp, appear to be critical for element transposition and expression. *Mutator* TIRs contain binding sites for the transposase where MURA protein was shown to bind a conserved ~32 bp sequence motif in active *Mutator* elements in maize [15]. In addition, two convergent genes contained within the maize autonomous *MuDR* element, including the transposase MURA, are transcribed from promoters located within the TIRs [16]. Furthermore, the *MuDR* TIR contains plant cell-cycle enhancer motifs which program a 20-fold upregulated expression in reproductive organs as compared with leaves [16]. The promoters in the TIRs are also responsible for the expression of the internal regions of Pack-MULEs [8], suggesting the importance of the TIR sequence for transposition and retention of MULEs in the genome. In this study, we report the identification and characterization of MULEs and Pack-MULEs with multiple TIRs in plants and the possible role of tandem TIRs in element amplification.

## 2. Methods

### 2.1. Plant Genomic Sequences and Construction of the Tomato MULE TIR Library

The sequence for the tomato (*Solanum lycopersicum*) genome was downloaded from the International Tomato Genome Sequencing Consortium. The sequence (http://www.solgenomics.net/organism/Solanum_lycopersicum/genome/, release 2.40). The sequence for rice (*Oryza sativa* ssp. *japonica* cv. Nipponbare) pseudomolecules was downloaded from the rice annotation group at Michigan State University (http://rice.plantbiology.msu.edu/, release 6.0). Maize (*Zea mays* cv. B73) chromosome sequences (4a.53) were downloaded from the maize sequencing project (http://www.maizesequence.org/, B73 RefGen_v1 [17]). The sequence for potato (*Solanum tuberosum *cv. DM) was downloaded from the Potato Genome Sequencing Consortium (http://potatogenomics.plantbiology.msu.edu, release 3.0 [18]). The *Arabidopsis* genome sequence (TAIR10) was downloaded from the *Arabidopsis* Information Resource (http://www.arabidopsis.org/). For identifying elements with additional TIRs, MULE TIR libraries that were generated previously were used for rice, maize, and *Arabidopsis* genomes [19].

The tomato MULE TIR library was built with an iterative process that uses Pairwise Alignment of Long Sequences (PALS 1.0) [20] to identify long inverted repeats (minimum length = 100 bp; minimum similarity = 80%). A custom python script was used to identify pairs of inverted repeats from the output of PALS, extract flanking sequences, and identify a 9–11 bp TSD. Manual curation was done to verify terminal inverted pairs with overall high sequence similarity in at least 100 bp sequence and having intact TIR ends and presence of a 9–11 bp TSD immediately flanking the ends of the TIR. Pairs that passed the above criteria were added to a TIR sequence library of tomato which was later filtered for redundancy. If two TIR sequences share 80% or higher similarity in at least 80% of their length, the two sequences are considered redundant, and one of the sequences is excluded. Among a redundant group of TIR sequences, the TIR sequence from the element with the highest TIR identity is retained in the nonredundant library.

### 2.2. Estimation of MULE Copy Number and Identification of Elements with Multiple TIRs from Plants

To determine the abundance of MULEs related to different TIR families (Table 1), copy number was estimated by considering one pair of TIRs as one element. To estimate how many elements are associated with a TSD, the presence of a TSD was verified using a pipeline consisting of perl scripts that search for 9-10 bp direct repeat with no more than 2 mismatches flanking the ends of the TIR sequences. The copy number of autonomous MULE elements was estimated from all elements retrieved from the previous step having significant match to known MULE transposases (*E *= 10^−5^, BLASTX) after filtering for low complexity.

To search for MULEs with multiple TIRs, elements from each genome sequence were identified using RepeatMasker (using default parameters; http://repeatmasker.genome.washington.edu/) with the rice, maize, tomato, and *Arabidopsis* MULE TIR libraries. A custom python script was used to identify elements flanked by 2 similar TIRs on one or both sides of the element. The following criteria were used to filter the results: (1) distance between the external TIRs is not larger than 20 kb and there is no sequencing gap between the TIRs, (2) TIRs must be at least 50 bp long, (3) truncations at the external ends of TIRs must be no more than 15 bp, (4) the two TIRs on one or both ends are less than 600 bp apart, and (5) presence of a 9–11 bp TSD with no more than 2 mismatches. Custom perl and python scripts were used in combination to extract the sequences of putative multiple TIR elements and their flanking sequences, and all elements were manually verified for the presence of a TSD. To define whether an element has multiple copies in a genome, the following criteria were applied: for individual elements, if the TIRs of two elements (with different TSDs) can be aligned (BLASTN, *E* = 10^10^), and if >70% of the sequence between the TIRs can be aligned (BLASTN, *E* = 10^−10^), then the two elements are defined as copies.

To obtain an approximate location of the elements in the tomato chromosomes, their coordinates in the pseudomolecules (release 2.4) were used to find nearby flanking SGN-markers as indicated in the Tomato Genome Browser (http://www.solgenomics.net/). The tomato FISH map was used as the basis of chromosome structure indicating centromere, euchromatin, and heterochromatin regions of each chromosome [21, 22]. The relative position of the flanking SGN-markers were identified in EXPEN-2000 physical map [23, 24] which have been linked to the FISH map by sequenced BACs. These maps are available through the Sol Genomics Network.

### 2.3. Phylogenetic Analysis of TIRs and Internal Sequences

To generate multiple sequence alignments, 220 bp of the external and internal TIRs at both ends (Figure 2) of the PM-ZIBP elements (the element containing a gene fragment from a gene encoding a zinc-ion binding protein, see Section 3) were used and resolved into lineages by generating phylogenetic trees. Multiple sequence alignment was performed by CLUSTALW (http://www.ebi.ac.uk/clustalw/) with default parameters. Phylogenetic trees were generated on the basis of the maximum likelihood method [25] with Kimura-2 parameter distances [26] using the MEGA 4 program (http://www.megasoftware.net/). Support for the internal branches of the phylogeny was assessed using 1000 bootstrap replicates. To compare the TIR of type 2-46 elements (see Section 3), 130 bp of the external and internal TIRs of type 2-46 elements were used to generate sequence alignment and a phylogenetic tree employing the same parameters and methods as the TIR comparisons for PM-ZIBP. Similar methods and parameters were used to generate sequence alignment and a phylogenetic tree for the acquired fragments in PM-ZIBP, the parental gene (SGN-U574419) in tomato, and gene sequences from potato, tobacco, and pepper.

### 2.4. Annotation of Pack-MULEs and Frequency of Element Sizes in Tomato

The procedure for the annotation of Pack-MULEs in the tomato genome was similar to that described previously [13]. Candidate Pack-MULEs and MULEs with multiple TIRs identified in this process were masked with all available tomato repeat sequences using RepeatMasker (http://repeatmasker.genome.washington.edu/, version open-3.0) with default parameters. The repeat library was built by combining repeats collected previously [27] with sequences matching transposase sequences in the RepeatMasker package (version open-3.0). The masked outputs were queried against the Solanaceae Unigene database (http://www.solgenomics.net/, version 5, BLASTN *E* = 10^−10^) and the nonredundant protein database in NCBI (http://www.ncbi.nlm.nih.gov/Blast.cgi *E* = 10^−05^) to identify gene fragments inside elements. To estimate element size, the elements were masked with all available tomato repeat sequences excluding MULE-related sequences using RepeatMasker to identify nested insertion of other transposons inside Pack-MULEs. The element size was then calculated using a custom perl script which excluded the masked sequence inside Pack-MULEs. Elements larger than 2 kb were not plotted due to their low abundance.

### 2.5. TIR Sequence Analysis and Conservation Test

The external and internal TIR sequences were compared using the “gap” program available from the GCG package (version 11.0, Accelrys Inc., San Diego, CA) to identify repeats found in the sequences. To determine the conservation of the TIR at nucleotide level, the first 700 bp sequence of each element was extracted and compared using DIALIGN2-2 [28] with default parameters. The normalized local sequence similarity scores as determined from DIALIGN2-2 were then used to determine an average similarity score for every 5 nucleotides and then plotted.

## 3. Results

### 3.1. Types of MULEs with Multiple TIRs

**Figure 1:**
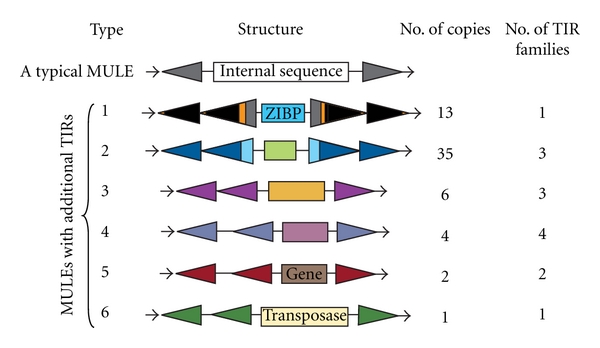
The structure of distinct types of MULEs with additional TIRs in tomato including their copy numbers and the number of TIR families involved in formation. Black horizontal arrows indicate target site duplication (TSD); solid colored triangles indicate Terminal Inverted Repeat (TIR); colored boxes indicate internal sequence and are labeled accordingly if sequences are annotated as genes or have similarity to MULE transposases.

A typical MULE contains one pair of TIRs, which refers to similar or identical terminal inverted sequences found on the opposite ends of the element (Figure 1, see above). In this study, we detected some atypical MULEs with two pairs of TIRs (type 1 and type 2, Figure 1), which will be referred to as external TIR and internal TIR, respectively. The external and internal TIRs have extended sequence similarity in at least 100 bp of the TIR region. In some cases, there is only one additional terminal sequence instead of one pair of terminal sequences (type 3, 4, 5, and 6, Figure 1), and this additional terminal sequence will be called a solo TIR to distinguish it from paired TIRs. Analysis of the tomato genome sequence revealed the presence of 61 MULEs with at least one additional TIR. All these elements are associated with a distinguishable TSD, a hallmark of transposition, suggesting that these elements are derived from transposition and not recombination. Furthermore, there is no recognizable TSD flanking the internal TIR, indicating that these elements are not formed through nested insertion of the same type of elements.

As shown in Figure 1, the majority of these elements (48 out of 61 elements, 79%) are type 1 and type 2 which contain external and internal TIRs located in tandem on both ends of the element (Figure 1). Some elements carry recognizable gene fragments (type 1 and type 5) and are therefore classified as Pack-MULEs. Thirteen elements (out of 61, 21%) are associated with one additional solo TIR only on one end of the element (type 3) or in the internal region (type 4, 5, and 6). Elements with tandem TIRs are more abundant than elements containing a solo TIR (48 versus 13). Three elements (out of 6) with tandem TIRs have multiple copies (elements are considered to be copies if they have similar TIR and similar internal region). The element with the highest copy number is flanked by two SLMULE46 TIRs (29 copies, type 2, and will be referred to as type 2-46 element), followed by a second group, an element with 13 copies that makes up the type 1 class. In contrast, among the 12 distinct elements containing one additional solo TIR, only one has another copy in the genome, and this element belongs to type 3 with SLMULE46 TIR. In fact, this element is the 3-TIR version of type 2-46 because it has a similar internal region (see Sections 3.3 and 4). This suggests that elements with solo TIRs may be dramatically less competent in transposition than elements with tandem TIRs on both ends.

In tomato, a total of 59 MULE TIR families have been identified with approximately 28,000 total copies of MULEs (see Table 1 for distribution of copies in each family). Among them, 10 TIR families (17%) are involved in the formation of elements with additional TIR sequences (Figure 1, Table 2). These 10 families have moderate copy numbers and a fraction of putative autonomous elements comparable to many other TIR families that are not associated with the formation of elements with multiple TIRs (Table 1). Eight families are represented in the formation of elements with solo TIRs, yet only three TIR families are involved in the formation of elements with tandem TIRs (type 1 and type 2, Table 2). In addition, most of the elements with this atypical TIR feature are non-autonomous elements in that they do not encode the proteins essential for transposition. The only exception is an element with an additional solo TIR, whose internal region was found to have sequence similarity to MULE transposases (type 6 in Figure 1). However, a close examination indicates the presence of numerous premature stop codons and frame-shifts in the coding region, suggesting the element is unlikely a currently functional autonomous element.

A similar analysis of rice and maize genomes to detect elements with multiple TIRs identified a few elements compared to the tomato genome (Tables 3 and 4). Two elements belonging to the type 3 and four elements (type 3 and 4) were uncovered in rice and maize genomes, respectively. In these two genomes, the presence of tandem TIR on both ends (type 1 or type 2) was not detected. In contrast, in *Arabidopsis*, a species that has previously been reported as having few MULEs and Pack-MULEs compared to rice and maize [17, 19, 29], eleven elements with tandem TIRs on both ends were found, including one Pack-MULE. This suggests that the formation of tandem TIR elements is not related to the abundance of MULEs and Pack-MULEs in the genome, and dicot genomes harbor more tandem TIR elements than genomes of monocots.

### 3.2. A Pack-MULE Family with Tandem TIRs

**Figure 2:**
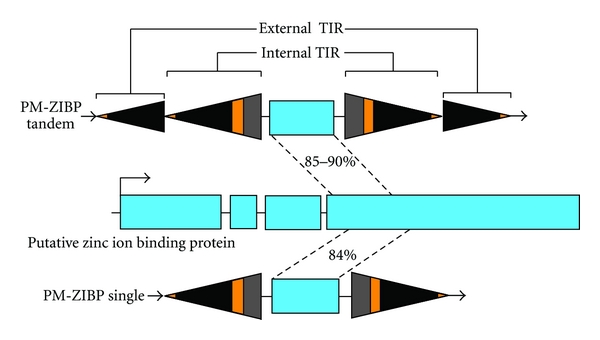
PM-ZIBP elements with single and tandem TIRs that contain a gene fragment. Solid triangles indicate TIRs and blue boxes indicate exons of gene SGN-U574419 and fragment acquired by the Pack-MULE. Introns are depicted as lines connecting exons.

**Figure 3:**
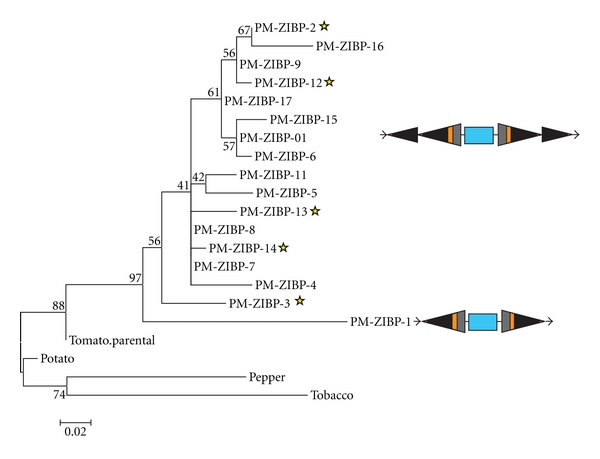
Phylogenetic analysis based on the acquired fragment in PM-ZIBP from SGN-U574419 and related sequences (SGN-U273862, SGN-U20267, and SGN-U506815). Sequences were aligned using ClustalW and phylogenetic reconstruction used the maximum likelihood method with Kimura-2 parameter distances implemented in the MEGA program. Bootstrap values are indicated as a percentage of 1000 replicates (40% majority rule consensus). Elements mapping to heterochromatic regions are indicated by a star symbol.

The elements comprising type 1 elements in tomato are copies of a Pack-MULE that harbors a fragment from a gene encoding a zinc-ion binding protein (Figure 2). This Pack-MULE family has 13 copies with tandem SLMULE18 TIRs located on both ends of the element and a single copy with one pair of TIRs (Figure 2), resembling that of a typical Pack-MULE. This family will be referred to as PM-ZIBP hereafter (Table 5). To dissect the relationship between the single TIR element and elements with tandem TIRs, a phylogenic tree was built using the acquired region, the parental gene in tomato, and the corresponding regions from other related plants including potato, pepper, and tobacco. The PM-ZIBP elements are grouped with the putative parental gene from tomato. Moreover, related elements are not present in the genome of potato suggesting that the acquisition of the gene fragment and the formation of the Pack-MULE may have occurred after the divergence of tomato and potato. Among the PM-ZIBP elements, the element with a single TIR (PM-ZIBP-1) forms a branch with the longest length (Figure 3). If the mutation rate is comparable among this group of elements, this implies that PM-ZIBP-1 is the most ancient element that acquired this gene fragment (or the ancestor of this element acquired the gene fragment), and elements with tandem TIRs are putative derivatives of PM-ZIBP-1. This is consistent with the fact that PM-ZIBP-1 is associated with the lowest TIR identity (Table 5), since young elements often have identical or highly similar TIRs. Our results show no evidence that elements with tandem TIRs are capable of acquiring gene sequence.

**Figure 4:**
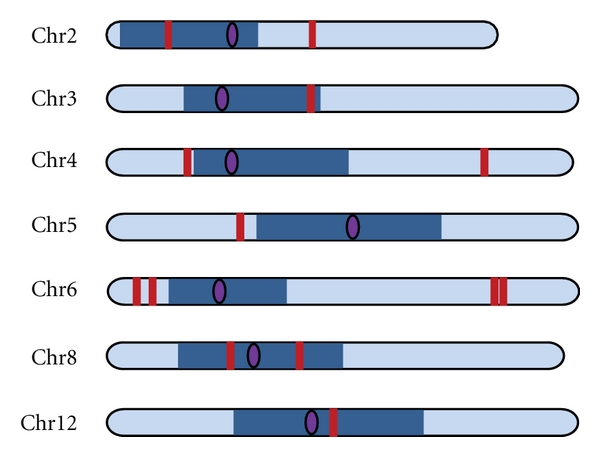
Chromosomal distribution of tandem TIR PM-ZIBP in tomato. Dark blue blocks represent heterochromatin and light blue regions represent euchromatin. Individual elements are represented by dark red vertical bars, and the purple ovals indicate the location of the centromere.

The 14 copies of PM-ZIBP were mapped onto the tomato chromosomes (Table 5). All the elements with tandem TIRs are mapped to distinct chromosomal loci with most of them in euchromatic regions. However, the single-TIR copy mapped to Chromosome 0 which consists of sequenced fragments that cannot be physically mapped to any of the chromosomes. Sequence analysis of the contig containing this element showed that it was highly repetitive suggesting its location in heterochromatic regions of the genome. This raised the question as to whether the long branch length associated with PM-ZIBP-1 element is an artifact of accelerated mutation in heterochromatic regions. To test this notion, the five elements with tandem TIRs that are located in heterochromatic regions (Figure 4) were examined, and these elements were found to be associated with both long and short branches (Figure 3). Moreover, none of them has a branch that is longer than that of PM-ZIBP-1. As a result, the location of PM-ZIBP-1 does not fully explain its branch length, and it is likely the oldest PM-ZIBP element. However, we cannot rule out the possibility that there are other unknown factors responsible for the unusually high mutation rate (in both TIR and internal regions) in PM-ZIBP-1 that are not correlated with its age. Despite this limitation, it is obvious that PM-ZIBP-1 has been present in the genome for a substantial amount of time, without being amplified.

### 3.3. Sequence Features with Elements Carrying Multiple TIRs

**Figure 5:**
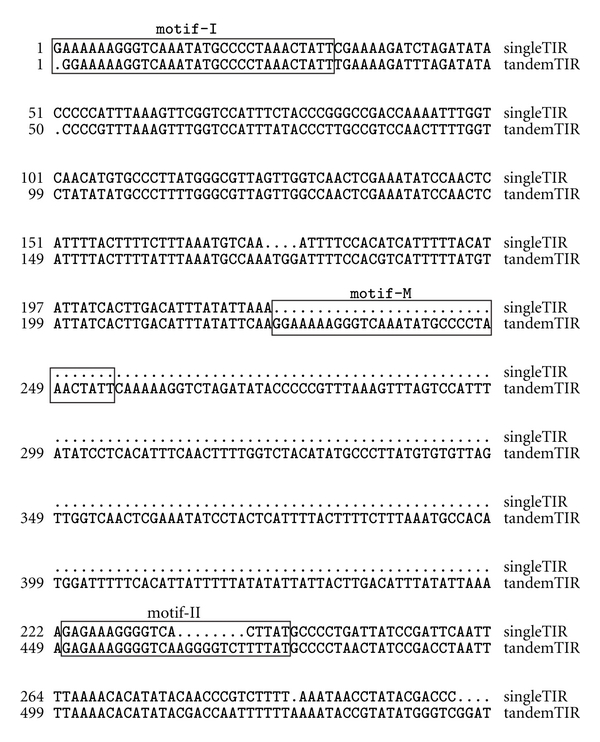
Alignment of TIR sequences from a PM-ZIBP with tandem TIRs and that from PM-ZIBP-1 illustrating the location of the 3 repetitive motifs found in the TIRs.

**Figure 6:**
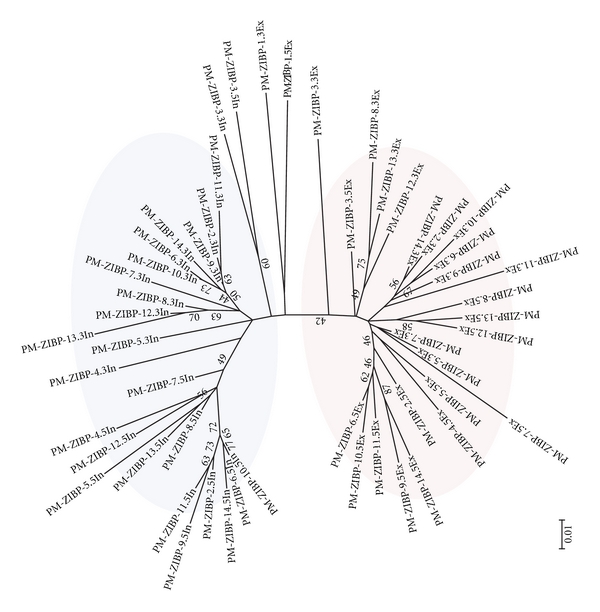
Phylogenetic analysis of sequences of the external, and internal TIRs of PM-ZIBP elements. TIR end is indicated by 5′ for TIR sequences from the left end and 3′ for TIR sequences from the right end. Sequence names ending in “Ex” indicate external TIRs while sequence names ending in “In” indicate internal TIRs. Sequences were aligned and phylogeny was reconstructed as described for Figure 3.

To understand the mechanism involved in the formation of PM-ZIBP elements in tomato, careful analysis, and comparison between the external and internal TIR sequences were performed for the single and tandem TIR copies. Three motifs with high sequence similarity were found in PM-ZIBP with tandem TIRs and two of these, motif-I and motif-II, were also found in the single TIR of PM-ZIBP-1 (Figure 5). The presence of the repetitive motifs in the TIR suggests that the additional TIR could be formed via a DNA replication slippage process involving a single TIR element. According to this model, when the replication proceeds to motif-II, the DNA polymerase slips from the DNA template and subsequently reattaches at motif-I so that the sequence between motif-I and motif-II is duplicated. If this is the case, the external and internal TIR should originate from the same template. To test this notion, phylogenetic analysis of the internal and external TIRs from the tandem TIR PM-ZIBP and the TIR of the single TIR element from both 5′ end and 3′ end was performed using ClustalW and MEGA (Figure 6). This analysis demonstrates a separate grouping of the internal, external and single TIRs, which seems to contradict with the slippage hypothesis. However, this is not definitive evidence against the slippage hypothesis since the bootstrap values are relatively low and separation of the external and internal TIR could be due to their distinct role in transposition (also see below).

**Figure 7:**
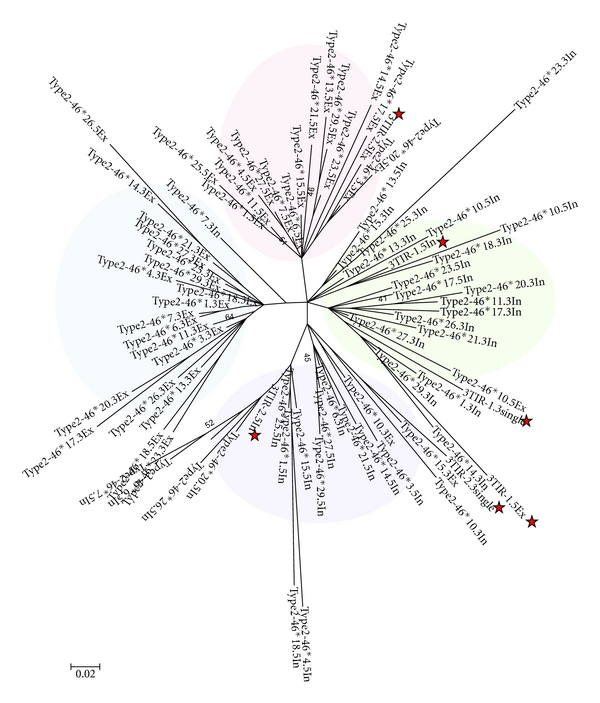
Phylogenetic analysis of external and internal TIRs from type 2-46. Naming of TIRs is similar to that for Figure 6 and alignment and phylogeny was reconstructed as described for Figure 3. TIRs from the 3-TIR elements are indicated by a star symbol.

The examination of another element with tandem TIRs (type 2-46) failed to identify the presence of similar motifs, suggesting that the presence of recognizable repetitive motif is not essential for the formation of tandem TIRs. Phylogenetic analysis between the external and internal TIRs of this family showed four fundamental groups (5′ internal, 5′external, 3′ internal, 3′ external) (Figure 7) with distinct branch lengths, suggesting they have been amplifying for an extended period. However, there are several TIRs intermingled with other groups and the bootstrap values for the major branches are rather low. As a result, it is difficult to make a clear-cut interpretation about the origin of the TIR duplication in this family. Interestingly, the two 3-TIR elements do not form an independent group. Instead, their TIRs intermingled with different elements with tandem TIRs. The branch length of the 3-TIR elements is comparable to other type 2-46 elements so they might have formed within a similar time-frame.

An alternative mechanism for the formation of elements with tandem TIRs is through recombination between elements with related TIRs. If this is the case, the initial parental elements are not expected to harbor a TSD. To test this hypothesis, we screened all 3-TIR elements and tandem TIR elements that are not associated with a TSD. A closer examination of these candidates indicates all of them have a certain level of truncation at the very termini of the TIR. As a result, it is not clear whether the lack of TSD is due to recombination or due to truncation. Thus, it remains an open question whether recombination played a role in the formation of these elements.

### 3.4. The Putative Role of the Tandem TIRs in Amplification of the Elements

**Figure 8:**
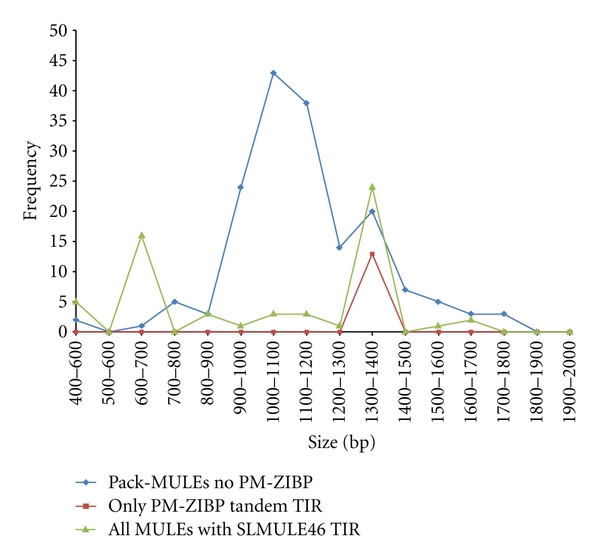
The frequency of different element sizes. Elements that are less than 2 kb are plotted.

As mentioned above, the Pack-MULE element with single and tandem TIR PM-ZIBP copies share terminal and internal sequences, yet the elements with tandem TIRs have many more copies than the single TIR PM-ZIBP-1 (13 versus 1). For the type 2-46 element with SLMULE46 TIRs, we failed to identify a corresponding element with single TIR and exactly the same internal sequence. However, other non-autonomous MULEs with single SLMULE46 TIR and associated with a TSD were identified and the copy number of none of them is as high as that of type 2-46 (29 copies). The copy numbers of these elements range from 1 to 14, with an average of 3 copies. A parsimonious explanation for such phenomenon is that the presence of the second TIR confers some advantage for transposition. The internal TIR may simply function as a filler DNA that allows the element to achieve an optimum size. To evaluate the role of size in transposition efficiency of PM-ZIBP, the distribution of Pack-MULE sizes in tomato was examined to identify a range that would correspond to optimal sizes for successful movement and amplification in the genome. The Pack-MULEs in tomato were grouped according to size at 100 bp increments. As shown in Figure 8, Pack-MULEs are most abundant with size ranging from 1000–1200 bp which is very close to that of the single TIR PM-ZIBP-1 (944 bp). There are elements from 9 TIRs with 24 different types of internal regions so the presence of this maximum is not due to the amplification of one or two element families. Meanwhile, a minor maximum was observed at 1300–1400 bp (composed of seven TIR families with 10 different internal sequences), which coincides with the size of PM-ZIBP associated with tandem TIRs. Interestingly, the sizes of the elements with tandem SLMULE46 TIRs that predominantly compose the type 2 elements (type 2-46) fall into the same peak as the PM-ZIBP with tandem TIRs (Figure 8). The presence of tandem TIRs (over 1 kb in total) and internal sequence makes it highly unlikely for a single element to be within 1000–1200 bp in size. As a result, 1300–1400 bp could be the optimal size for elements with tandem TIRs, regardless of the presence or absence of gene fragments in the internal region.

**Figure 9:**
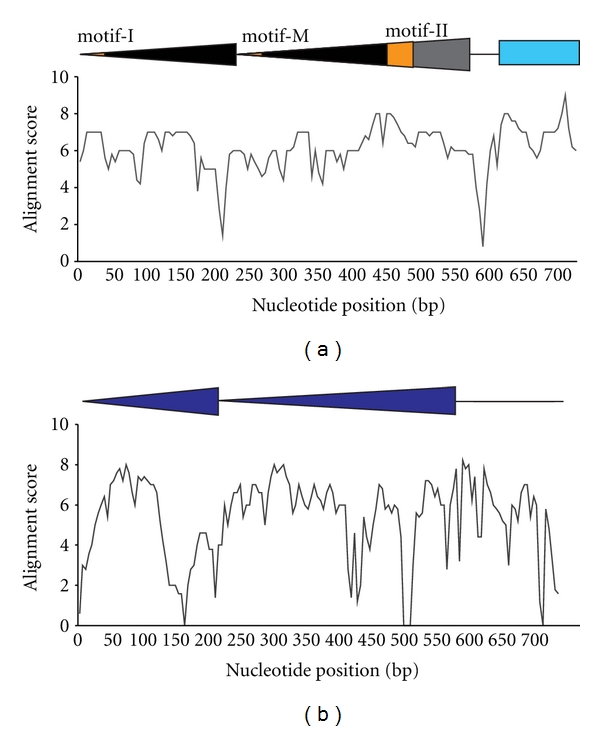
Nucleotide conservation across the two tandem TIRs. (a) Tandem TIRs from PM-ZIBP. (b) Tandem TIRs from type 2-46. The nucleotide conservation scores are calculated as an average of 5 nucleotide position scores from the copies of the element. Colored or black triangles represent the TIR. In Figure 9(a), the orange regions indicate the 3 repetitive motifs (see text). Colored box indicates part of the acquired gene fragment.

An additional explanation for the abundance of tandem TIR elements over their single TIR counterpart is an advantage conferred by the tandem TIR resulting in increased frequency of recognition by the transposase or enhanced interaction between the element and the transposition machinery. If this is the case, one would expect significant sequence conservation in both TIRs. Comparison of sequence identity between the external TIRs and the internal TIRs of PM-ZIBP shows that the initial part of the internal TIR is slightly less conserved compared to that of the external TIR. However, the majority of the TIR sequence has a similar level of conservation, indicating that both TIRs may play functional roles (Figure 9(a)). The most conserved region is motif II and its adjacent region (orange and grey region, Figure 9(a)), which is not present in the external TIR, suggesting the importance of this region. This is in contrast to the low conservation level in regions between the TIR and the acquired gene fragment. Interestingly, the conservation level of the acquired gene fragment is comparable or slightly higher than that of TIRs, suggesting that the gene fragments might be functional. The divergence of the internal TIR around motif-M may be a result of selection to ensure that precise cleavage occurs in the external TIR instead of the internal TIR upon excision of the element. This is in concordance with the fact that no element with single TIR appears to be derived from elements containing tandem TIRs among PM-ZIBP elements.

Compared to the internal region, the TIRs of the type 2-46 elements have considerable level of conservation, which is similar to that of PM-ZIBP (Figure 9(b)). However, unlike the PM-ZIBP elements, the most internal region of the internal TIR does not demonstrate an elevated level of conservation, suggesting the variation in location of important *cis*-elements among different families of TIR sequence. Furthermore, a low level of conservation was observed in the internal region of this group of elements, which is consistent with the lack of gene fragments in its internal region.

## 4. Discussion

### 4.1. The Formation and Amplification of MULEs with Additional TIR Sequence

The TIR sequences of DNA elements contain *cis*-elements that are responsible for interaction with and recognition by the relevant transposases. It also contributes to the selection of insertion site as well as serving as the target for epigenetic regulation [4, 30]. As a result, the TIR sequences play a critical role in the successful amplification of relevant TEs. For many DNA transposons, short repetitive motifs are present in the TIR or sub-terminal regions, either in direct or inverted orientations. Nevertheless, the duplication of an entire TIR (or almost an entire TIR) is unusual and has not been studied previously. In this study, 59 MULE TIR families in tomato were examined and 10 of them are associated with TIR duplication. This indicates that certain MULE TIR families have a propensity to form duplicate TIRs over others, and the frequency is not correlated with the total copy number of the particular TIR family. Among the elements with multiple TIRs, some are only associated with one solo TIR (type 3, 4, 5, and 6, with 8 TIR families) and others are associated with duplicated TIRs on both ends (type 1 and 2, with 3 TIR families). Obviously, few TIR families are associated with the formation of duplicated TIRs on both ends, suggesting this is a less frequent event. However, only one element containing a solo TIR has an additional copy, and it is uncertain whether the two copies are derived from each other (see Section 4.2). This suggests the destiny of “death on birth” for elements with a solo TIR. It is possible that the presence of one additional TIR resulted in a lack of structural symmetry which interferes with transposition. In other words, the presence of one additional TIR sequence could have negative impact on transposition competency. In contrast, the elements with tandem TIRs on both ends are more successfully amplified, despite their low frequency of initial formation.

### 4.2. The Mechanism Involved in the Formation of Duplicated TIRs

At present it is not clear how the TIR sequence was duplicated in these atypical MULEs. DNA replication slippage is considered a common mechanism to cause deletion or duplication of sequences when repetitive motifs are present in adjacent regions. This seems to apply to the PM-ZIBP elements due to the presence of repetitive motifs inside the TIR sequence. Nevertheless, this hypothesis is not unambiguously supported by phylogenetic analyses of internal and external TIR sequences. Furthermore, not all elements with additional TIR have significant repetitive motifs inside the TIR. As a result, there may be other mechanisms involved in the duplication of TIRs. This may include duplication by recombination or through nested insertion followed by loss of TSD for the internal element. If recombination is the main factor that drives the formation of elements with multiple TIRs, one would expect those elements to be overrepresented among the TIR families with highest copy numbers in the genome. Nevertheless, it does not seem to be the case (Table 1).

Another question about the formation of elements with tandem TIR is whether the duplication of TIR at both ends is a single event or a step-wise process. Based on the fact that there are elements with 3 TIRs, it is possible that the duplication is a step-wise process. The coexistence of type 2-46 with two corresponding 3-TIR elements seems to suggest this is the case. However, the phylogenic analysis does not support the notion that the 3-TIR elements are older than all type 2-46 elements (Figure 7). Thus, it is unlikely that the 3-TIR elements are the direct progenitor of elements with tandem TIRs, and the true ancestor may have been lost from the genome. On the other hand, since the two 3-TIR elements are more closely related to type 2-46 elements than to each other, it seems to imply they are not derivatives of each other. In this case, an alternative scenario is that the two 3-TIR elements are derivatives of distinct type 2-46 elements through aberrant transposition. This may occur, for example, when one external TIR and one internal TIR in type 2-46 are recognized for transposition. This is consistent with the fact that none of the other 3-TIR elements has a duplicated copy, so the duplication of this particular 3-TIR element may have not arisen through the transposition of itself.

It is known that non-autonomous MULEs are capable of acquiring genomic sequences including genes. The frequency of acquisition of genes by MULEs seems to be higher than that of other DNA elements with shorter TIRs [17]. Moreover, the acquired sequences can be integrated into extended TIR sequences [13, 31]. Given this fact, it is conceivable that the additional TIR could also be introduced through acquisition. Unfortunately, the mechanism of sequence acquisition is yet to be understood.

The comparison of copy number of elements with tandem TIRs in different genomes may provide additional insights into this question. Considering the abundance of MULEs and Pack-MULEs in the genomes of maize and rice, it is striking that only a few MULEs with additional TIRs are found in these genomes. However, if we assume that tandem TIRs are formed through sequence acquisition, the phenomenon can be readily explained. The genomes of maize and rice contain substantially more GC-rich sequences (or a more significant GC gradient) than that of *Arabidopsis* [32, 33]. Pack-MULEs in rice and maize demonstrate a strong preference for acquiring GC-rich sequences [19, 34]. Since the GC content of Pack-MULE TIR is similar or lower than the genomic average level [19], the acquisition of additional TIR sequence would be discriminated against in the genomes of rice and maize due to their relatively low GC content compared to gene sequences. In contrast, the GC content of sequences in dicot genomes is less variable [19, 35], such that TIR sequences are more likely to be acquired than their counterparts in the genomes of monocots. This might explain why there are more elements with tandem TIRs in tomato and *Arabidopsis* than in maize and rice.

### 4.3. Possible Competency Conferred by Tandemly Duplicated TIR

Among the elements with duplicated TIRs, two tomato elements have amplified to a certain degree. The PM-ZIBP elements have 13 copies with an additional copy that is associated with only a single TIR. The type 2-46 elements have 29 copies without a corresponding copy with a single TIR, yet this particular TIR family is associated with single TIR elements harboring distinct internal regions with a lower copy number. Due to the coincidence of elements with tandem TIRs and single TIRs, it is clear that the presence of duplicated TIRs is not required for transposition, at least for these two TIR families. This raises the question whether the additional TIR has any role in transposition or successful amplification of these elements.

There are several explanations for the overrepresentation of elements with tandemly duplicated TIRs among the PM-ZIBP elements. Our analysis excludes the possibility that the duplicated TIR is acting as a filler DNA to allow the element to achieve an optimal size for amplification. It is worth pointing out that the “optimum size” might be present due to reasons other than size. If that is case, it also implies that the failure of PM-ZIBP-1 to amplify is unlikely attributable to its size. An alternative possibility points to the role of the internal region of PM-ZIBP since the acquired region appears more conserved than other internal sequence. According to this model, the PM-ZIBP is amplified because of the functional role of the acquired fragment. The element with single TIR (PM-ZIBP-1) failed to amplify due to its genomic location that is likely in heterochromatic region and not accessible for the transposition machinery. Nevertheless, due to the excision activity of DNA transposons and insertion polymorphism in the population, only a small subset of the transposons formed by transposition will be retained in the genome. If this model is valid, this may imply that PM-ZIBP-1 is the sole copy with a single TIR that has ever been present in the genome and one of the elements with duplicated TIRs must have been directly derived from PM-ZIBP-1. If this was the case, this would require PM-ZIBP-1 to be accessible in a certain way, which contradicts the original assumption of this model. Alternatively, there were other copies of PM-ZIBP with single TIR in the genomic location with more open chromatin that gave birth to the element with tandem TIRs. In either case, one or more of the elements with single TIR was in an accessible location but failed to amplify while their counterparts with tandem TIRs significantly increased their copy number. In addition, the type 2-46 element has amplified to 29 copies without an apparently functional internal region, suggesting that the presence of gene fragments is not required for the amplification of elements with duplicated TIRs. Taken together, the over-representation of PM-ZIBP and type 2-46 elements with tandem TIRs likely reflects an elevated competency for transposition for these two specific MULE families. This could be achieved by increased recognition of the element by the transposase and/or interaction with transposase. This is in accordance with the fact that sequence conservation was observed for both internal and external TIRs.

## 5. Conclusion

Transposable elements are the major components of plant genomes. MULEs play important roles in plant genome evolution due to their high activity and potential to acquire and amplify gene fragments. In this study, we uncovered that formation of duplicated TIRs might have contributed to the success of some specific MULE elements. The availability of genomic sequences from multiple plant genomes allows us to conduct a comprehensive analysis which led to the following conclusions: (1) the formation of elements with additional TIR is not a rare event but only elements with duplicated TIRs on both terminus have significant mobility; (2) the genome of dicots harbor more elements with duplicated TIRs than that of monocots, and such difference might be attributed to the presence of GC-rich sequences in the genomes of monocots; (3) distribution of size versus copy number of MULEs (or Pack-MULEs) is periodic, suggesting the distance between the TIRs or the relative spatial position of TIRs may have a role in transposition; (4) in the elements with tandem TIRs, both TIRs appear to be subject to certain constraints, and the presence of duplicated TIRs may confer certain mechanistic advantages for transposition. Such features may be utilized to create elements with elevated transposition activity.

## Figures and Tables

**Table 1:** Distribution of elements among tomato MULE TIR families.

TIR family	Copy number	No. with TSD	percentage with TSD	Autonomous elements	percentage of Autonomous
SLMULE56	8	1	12.5	0	0.0
SLMULE22	11	3	27.3	0	0.0
SLMULE28	13	4	30.8	0	0.0
SLMULE07	25	5	20.0	3	12.0
SLMULE48	26	7	26.9	0	0.0
SLMULE50	34	2	5.9	0	0.0
SLMULE49	40	1	2.5	2	5.0
SLMULE34	42	1	2.4	0	0.0
SLMULE25	49	13	26.5	9	18.4
SLMULE52	56	11	19.6	0	0.0
SLMULE44	62	2	3.2	1	1.6
SLMULE38	68	22	32.4	10	14.7
SLMULE31	71	5	7.0	3	4.2
SLMULE39	88	26	29.5	3	3.4
SLMULE54	100	28	28.0	12	12.0
SLMULE43	106	63	59.4	35	33.0
SLMULE47	109	25	22.9	0	0.0
SLMULE17	112	4	3.6	5	4.5
SLMULE45	112	12	10.7	2	1.8
SLMULE20	114	36	31.6	0	0.0
SLMULE53	116	8	6.9	3	2.6
SLMULE10	118	3	2.5	35	29.7
SLMULE11	130	43	33.1	7	5.4
SLMULE59	144	49	34.0	2	1.4
SLMULE42	156	6	3.8	1	0.6
SLMULE27	164	72	43.9	19	11.6
SLMULE29	164	64	39.0	1	0.6
SLMULE16	167	40	24.0	4	2.4
SLMULE30	176	31	17.6	1	0.6
SLMULE51	210	64	30.5	1	0.5
SLMULE23	233	84	36.1	1	0.4
SLMULE04	250	4	1.6	2	0.8
SLMULE58	262	66	25.2	2	0.8
SLMULE26*	266	56	21.1	0	0.0
SLMULE13	273	73	26.7	94	34.4
SLMULE55	275	87	31.6	2	0.7
SLMULE57*	337	178	52.8	10	3.0
SLMULE40	361	173	47.9	20	5.5
SLMULE36	373	100	26.8	17	4.6
SLMULE24*	381	96	25.2	4	1.0
SLMULE41	413	126	30.5	3	0.7
SLMULE46*	418	62	14.8	2	0.5
SLMULE32	444	199	44.8	62	14.0
SLMULE15	455	130	28.6	9	2.0
SLMULE18*	498	198	39.8	11	2.2
SLMULE03	546	193	35.3	10	1.8
SLMULE37*	635	361	56.9	40	6.3
SLMULE08*	644	133	20.7	4	0.6
SLMULE09*	760	425	55.9	26	3.4
SLMULE35	823	407	49.5	30	3.6
SLMULE14	1144	236	20.6	1	0.1
SLMULE21	1301	73	5.6	7	0.5
SLMULE33*	1310	548	41.8	16	1.2
SLMULE05*	1341	110	8.2	7	0.5
SLMULE06	1621	816	50.3	18	1.1
SLMULE02	1685	748	44.4	1	0.1
SLMULE12	1798	748	41.6	5	0.3
SLMULE19	2386	914	38.3	3	0.1
SLMULE01	4017	2609	64.9	3	0.1

Total	28041	10604	38%	569	2%

* MULE TIR families involved in TIR duplication.

**Table 2:** MULE TIR families involved in TIR duplication in tomato.

Type	MULE TIR	Number of copies
1	SLMULE18	13
2	SLMULE18	1
	SLMULE33	1
	SLMULE46	33
3	SLMULE26	1
	SLMULE46	4
	SLMULE57	1
4	SLMULE05	1
	SLMULE08	1
	SLMULE26	1
	SLMULE46	1
5	SLMULE09	1
	SLMULE24	1
6	SLMULE37	1

**Table 3:** List of the multiple TIR elements in *Arabidopsis*, maize, and rice.

Element ID	Genome	Chromosome	Position start	Position end	TIR family	Type*
AtMULEdt-1	*Arabidopsis*	1	15918026	15919043	At000317	2
AtMULEdt-2	*Arabidopsis*	1	16260326	16261097	At000824	2
AtMULEdt-3	*Arabidopsis*	2	5670143	5671281	At000317	2
AtMULEdt-4	*Arabidopsis*	2	6446333	6451005	At000317	1
AtMULEdt-5	*Arabidopsis*	3	16141980	16143074	At000317	2
AtMULEdt-6	*Arabidopsis*	4	1230491	1231738	At000800	2
AtMULEdt-7	*Arabidopsis*	4	6700242	6701357	At000800	2
AtMULEdt-8	*Arabidopsis*	4	2341448	2342412	At000317	2
AtMULEdt-9	*Arabidopsis*	5	9709514	9710845	At000800	2
AtMULEdt-10	*Arabidopsis*	5	17479616	17480806	At000317	2
AtMULEdt-11	*Arabidopsis*	5	18980159	18981488	At000800	2
OsMULEdt-1	Rice	1	20126005	20126814	Os0182	3
OsMULEdt-2	Rice	9	8210481	8216333	Os1455	3
ZmMULEdt-1	Maize	1	239115417	239115828	Zm15155	3
ZmMULEdt-2	Maize	5	17121166	17121599	Zm00411	4
ZmMULEdt-3	Maize	8	94462286	94472999	Zm28610	4
ZmMULEdt-4	Maize	8	121663900	121667746	Zm00411	4

*Based on the same classification as Figure 1.

**Table 4:** Frequency of MULEs, Pack-MULEs and MULEs with multiple TIRs in four different plant genomes.

Element type	Arabidopsis	Tomato	Rice	Maize
MULEs	1576	28041	30475	12900^a^
Pack-MULEs	46^b^ (2.92)	220 (1.6)	2853^b^ (9.4)	276^b^ (2.1)
MULEs with multiple TIRS	11 (0.70)	61 (0.22)	4 (0.01)	2 (0.02)

Number in parenthesis indicate percentage from total number of MULEs.

^a^Schnable et al., 2009 [17]

^b^Jiang et al., 2011 [19].

**Table 5:** List of the Pack-MULEs that captured a fragment of a putative zinc-ion binding protein.

Element ID	Chromosome	Position start	Position end	Size	TSD sequence	Percentage of outer TIR identity	Percentage of inner TIR identity
PM-ZIBP-1	0	12038207	12039150	944	TTTTAAATT	87	N/A
PM-ZIBP-2	2	29724087	29725458	1372	TAAATTATA	94	92
PM-ZIBP-3	2	33667391	33668737	1347	TACATTTTAA	92	90
PM-ZIBP-4	3	50199015	50200383	1369	TTAAAATTA	91	93
PM-ZIBP-5	4	4315606	4316970	1365	TATTATAAA	95	90
PM-ZIBP-6	4	58126752	58128120	1369	GTCAGGTTAA	93	91
PM-ZIBP-7	5	10380074	10381444	1371	ATAAAAGAT	93	92
PM-ZIBP-8	6	29844899	29846272	1374	CTTCGAGAC	91	92
PM-ZIBP-9	6	41871484	41872853	1370	TTTATTTAC	90	89
PM-ZIBP-10	6	42030690	42032062	1373	TTAAAAAAA	92	92
PM-ZIBP-11	6	7121877	7123250	1374	TTAAAAGAA	90	90
PM-ZIBP-12	8	14577081	14578450	1370	GAATAATAA	93	91
PM-ZIBP-13	8	4530978	4532348	1371	TTTTGGGAA	93	89
PM-ZIBP-14	12	9912832	9914208	1377	TATTTTTAT	92	90
